# Pruned U-net with multi-scale feature fusion and attention for real-time UAV remote sensing of levee defects

**DOI:** 10.1038/s41598-025-26431-0

**Published:** 2025-11-27

**Authors:** Bangbin Wu, Bo Chen, Xinxin Jiang, Zhi Liu

**Affiliations:** 1 Jiangxi Academy of Water Science and Engineering, Nanchang, 330029 China; 2 College of Water Conservancy and Hydropower Engineering, Hohai University, Nanjing, 210098 China; 3 College of Water Conservancy, Jiangxi University of Water Resources and Electric Power, Nanchang, 330099 China

**Keywords:** UAV remote sensing, Levee concrete defect detection, Deep learning, Semantic segmentation, Lightweight network, Engineering, Mathematics and computing

## Abstract

The long-term performance of levee infrastructure is increasingly threatened by environmental exposure and material degradation, underscoring the need for efficient, accurate inspection. Unmanned Aerial Vehicle (UAV)-based remote sensing offers a cost-effective solution, enabling rapid acquisition of high-resolution imagery over large surfaces; however, stains, occlusions, and illumination variability frequently degrade automated detection. To address these challenges, we propose a real-time semantic segmentation framework built on an optimized U-Net. The model integrates structured pruning to accelerate inference, a residual convolutional block attention module (ResCBAM) to suppress background interference and enhance defect saliency, and a multi-scale feature-fusion strategy with online feature distillation to strengthen fine-grained representations across resolutions. We evaluate the approach on UAV imagery collected from an aged levee section. The proposed method attains 90.05% accuracy, 88.94% recall, 89.22% precision, and 88.67% IoU, outperforming state-of-the-art baselines, while achieving a real-time processing rate of 57.74 FPS. These results demonstrate that the framework delivers a favorable speed–accuracy trade-off and is suitable for large-scale UAV-based levee monitoring. Overall, the experiments indicate strong potential for timely defect identification and proactive risk management in levee systems.

## Introduction

Infrastructure plays a pivotal role in the functioning of modern societies, encompassing a wide range of physical assets such as embankments, bridges, roads, tunnels, dams, power grids, and communication networks^1–3^. These systems are essential for transportation, energy supply, water management, and communication, forming the backbone of economic activity and quality of life^4^. However, the longevity and reliability of infrastructure are continuously challenged by aging, environmental factors, and increased demand^5^. As infrastructure ages, it becomes more susceptible to deterioration, requiring regular maintenance and timely intervention to prevent failure. Traditionally, infrastructure inspection has relied heavily on manual labor, involving costly and time-consuming processes, as well as significant risks to personnel^6^.

The rapid advancement of unmanned aerial vehicle (UAV) technology has revolutionized the field of infrastructure maintenance, offering a novel approach to the damage inspection of critical structures^7–10^. UAVs are particularly valuable for infrastructure maintenance because they can rapidly capture large volumes of data while minimizing human exposure to hazardous environments. The UAV-based damage inspection systems provide a safer, more efficient, and cost-effective alternative. These UAV-based systems are equipped with high-resolution cameras, sensors, and advanced imaging technologies, enabling precise data collection from difficult-to-reach areas^11^. Furthermore, the ability to operate autonomously and in real-time enhances both the frequency and quality of infrastructure inspections, leading to early detection of potential issues and reducing the likelihood of costly repairs or catastrophic failures.

In recent years, the integration of computer vision (CV) and artificial intelligence (AI) has further enhanced the capabilities of damage detection for civil infrastructures, enabling automated and highly accurate damage detection^12–17^. Convolutional neural networks (CNNs) and other DL-based techniques are particularly effective at recognizing subtle structural anomalies that may go unnoticed by human inspectors. For example, Li et al.^18^ developed a flexible crack identification system using a trainable context encoder network with neural blocks to enhance crack detection and segmentation. Liu et al.^4^ proposed a deep network combining 2D spatial and 3D geometric feature extraction to address low-contrast crack segmentation and multi-scene compatibility. The model achieved accurate pixel-wise segmentation, enabling precise crack measurement and analysis. Zhang et al.^19^ proposed a crack detection system using fusion features and incremental learning for efficient training without GPU acceleration, which achieved comparable accuracy to leading CNNs while improving training speed by over 20 times. From the above-mentioned literature, it can be inferred that these existing studies have contributed to damage detection in civil infrastructure, but there is still a lack of targeted research on damage detection in complicated UAV aerial inspection scenarios for water-related civil infrastructure. Firstly, UAV inspections are conducted outdoors, where lighting conditions, shadows, weather, and background noise like vegetation or debris may significantly impact image quality and damage detection accuracy. Low-contrast damages, glare, or uneven illumination exacerbate detection difficulties^20,21^. Moreover, UAVs often capture images from different altitudes and angles, leading to variations in resolution, perspective distortion, and scale differences. In addition, the limited computational resources available on UAVs demand damage detection algorithms with high computational efficiency to ensure real-time processing and reliable results during aerial inspections of civil infrastructure.

To improve the inference efficiency of damage detection models for UAV inspection scenarios, researchers have focused extensively on model lightweight techniques^22–24^. These methods aim to reduce the computational complexity of deep learning models while maintaining high detection performance, making them suitable for real-time processing on UAVs with limited computational resources. For example, Xu et al.^25^ proposed a lightweight semantic segmentation method for bridge damage recognition, enhancing the DeepLabv3 + model with the MobileNetV2 backbone and refining the atrous spatial pyramid pooling module, which achieved high accuracy with reduced parameters and recognition time. Ye et al.^26^ proposed a lightweight two-stage AI model for underwater structural damage detection, which achieved significant improvements in accuracy and efficiency, outperforming existing methods. The first stage enhances images using an improved CycleGAN and Retinex, while the second stage employs a lightweight YOLOv5 model for damage detection. Wang et al.^27^ proposed a lightweight crack segmentation network using knowledge distillation. The student model is trained with the pre-trained teacher model via channel-wise distillation and Kullback–Leibler divergence minimization to improve crack localization. In UAV-based remote sensing of levee infrastructures, lightweight models have been increasingly adopted to improve detection efficiency^28^. While such approaches accelerate inference, they often compromise robustness when confronted with complex noise and heterogeneous field conditions. A central challenge, therefore, lies in achieving a balance between real-time efficiency and reliable defect recognition under diverse disturbances^29–31^. Effective methods must be capable of handling environmental variability, changes in imaging perspective, motion artifacts, and limited computational resources, thereby ensuring accurate and consistent detection in large-scale UAV inspection of levee surfaces.

To address the above challenges, this study proposes a real-time, lightweight damage detection framework tailored for UAV-based remote sensing of levee infrastructures. A U-Net-based semantic segmentation network is developed and pruned to accelerate inference while maintaining efficiency. To improve robustness in complex field environments characterized by stains, vegetation occlusion, and illumination variations, an enhanced residual convolutional block attention module (ResCBAM) is incorporated, enabling more reliable discrimination of true structural defects. In addition, the integration of online feature distillation with a multi-scale feature fusion strategy strengthens the representation of fine-grained defect patterns across different resolutions. These improvements collectively enhance the performance of the lightweight model, ensuring accurate and robust defect recognition for large-scale levee monitoring.

The main contributions of this study can be attributed as follows.

1. A lightweight U-Net–based semantic segmentation model was developed and structurally pruned to enable real-time UAV monitoring of levee surfaces, achieving efficient inference with significantly reduced computational and memory costs.
2. An enhanced ResCBAM attention module combining both channel and spatial mechanisms was introduced to improve the recognition of levee surface defects under challenging conditions such as stains, vegetation occlusion, and illumination variations.
3. An online feature distillation framework integrated with multi-scale feature fusion was designed to align structural representations across resolutions, thereby enhancing the accuracy and robustness of UAV-based levee defect detection.

The remainder of this paper is organized as follows. “Methodology” details the proposed lightweight U-Net framework, including structured pruning for efficiency, an enhanced ResCBAM attention module, online feature distillation, and a multi-scale feature fusion scheme. “Case study” outlines the engineering context and experimental protocol, covering the levee-UAV dataset, evaluation metrics, and implementation specifics. “Experimental result and discussion” reports and analyzes the results, highlighting detection accuracy, robustness under complex field conditions, and comparisons with state-of-the-art baselines. Finally, “Conclusions and discussion” concludes and sketches directions for future research.

## Methodology

### The lightweight U-Net-based network optimized by model pruning

**Fig. 1:**
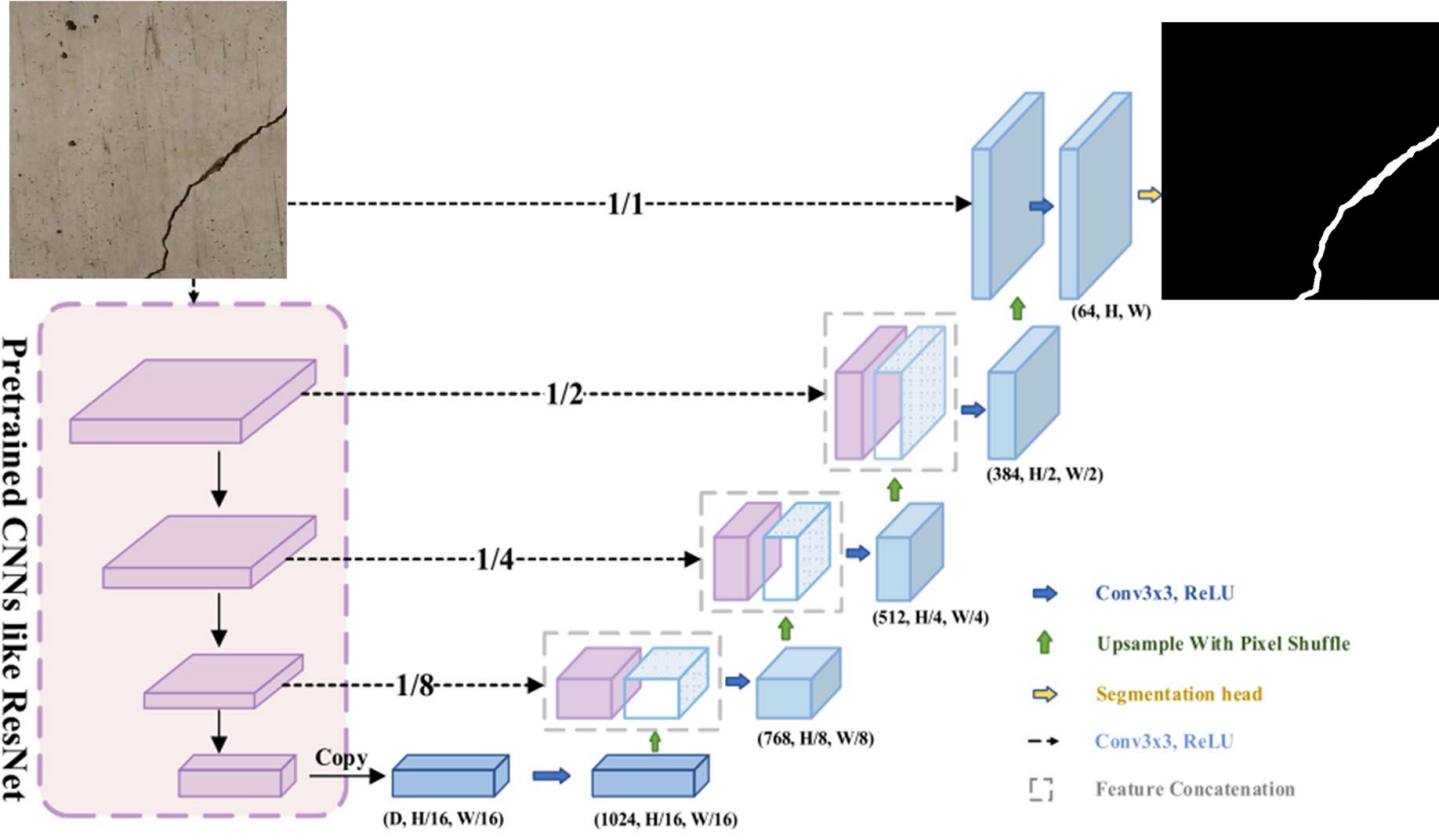
The U-Net-based network for crack pixel-wise segmentation.

U-Net is a convolutional neural network architecture primarily designed for semantic segmentation tasks. Figure 1 demonstrates the diagram of the U-Net-based network for crack pixel-wise segmentation. Its key feature is the encoder-decoder structure with skip connections, which enables the model to efficiently capture both low-level features (from the encoder) and high-level contextual information (from the decoder) to make pixel-wise predictions. This architecture allows the U-Net to produce accurate segmentation maps by maintaining fine-grained spatial details while progressively refining the feature maps.

U-Net, while effective for crack detection, has several limitations, particularly related to its computational complexity. The deep architecture and pixel-wise predictions result in high memory usage and processing time, which can be problematic during both training and inference, especially with large-scale datasets or high-resolution images from UAVs^32^. Additionally, the large number of parameters in the encoder-decoder structure leads to a substantial model size, which may limit its use in real-time applications on resource-constrained devices. Furthermore, due to their complexity, U-Net models are prone to overfitting, especially when training data is limited or lacks variability, which can impact their ability to generalize to new or unseen cracks.

In the DL-based semantic segmentation algorithm, the presence of multiple layers of nonlinear transformations often results in an immense computational burden, especially when handling large-scale data^33^. The computational complexity is exacerbated during both the training phase, which involves large matrix operations for backpropagation, and during inference in real-world applications, where the computational resources required for forward propagation can be substantial. This challenge becomes particularly acute in the context of UAV-based infrastructure inspection, where high-resolution images or videos of infrastructure structures are processed. The computational complexity increases when dealing with high-dimensional data and sophisticated models, which are common in UAV-based infrastructure inspection tasks^34^.

In such scenarios, optimizing DL models to handle these challenges efficiently becomes essential. The use of techniques such as model pruning, including global-local channel pruning, offers a solution to reduce the computational burden without sacrificing model performance^35^. By pruning less significant channels and reducing redundant computations, UAV-based infrastructure inspection systems can operate more efficiently, allowing for faster analysis of large-scale datasets collected from UAVs. This optimization is particularly important when operating in resource-constrained environments, such as on-board processing units or real-time systems used in UAVs, where the processing power is limited.

**Fig. 2:**
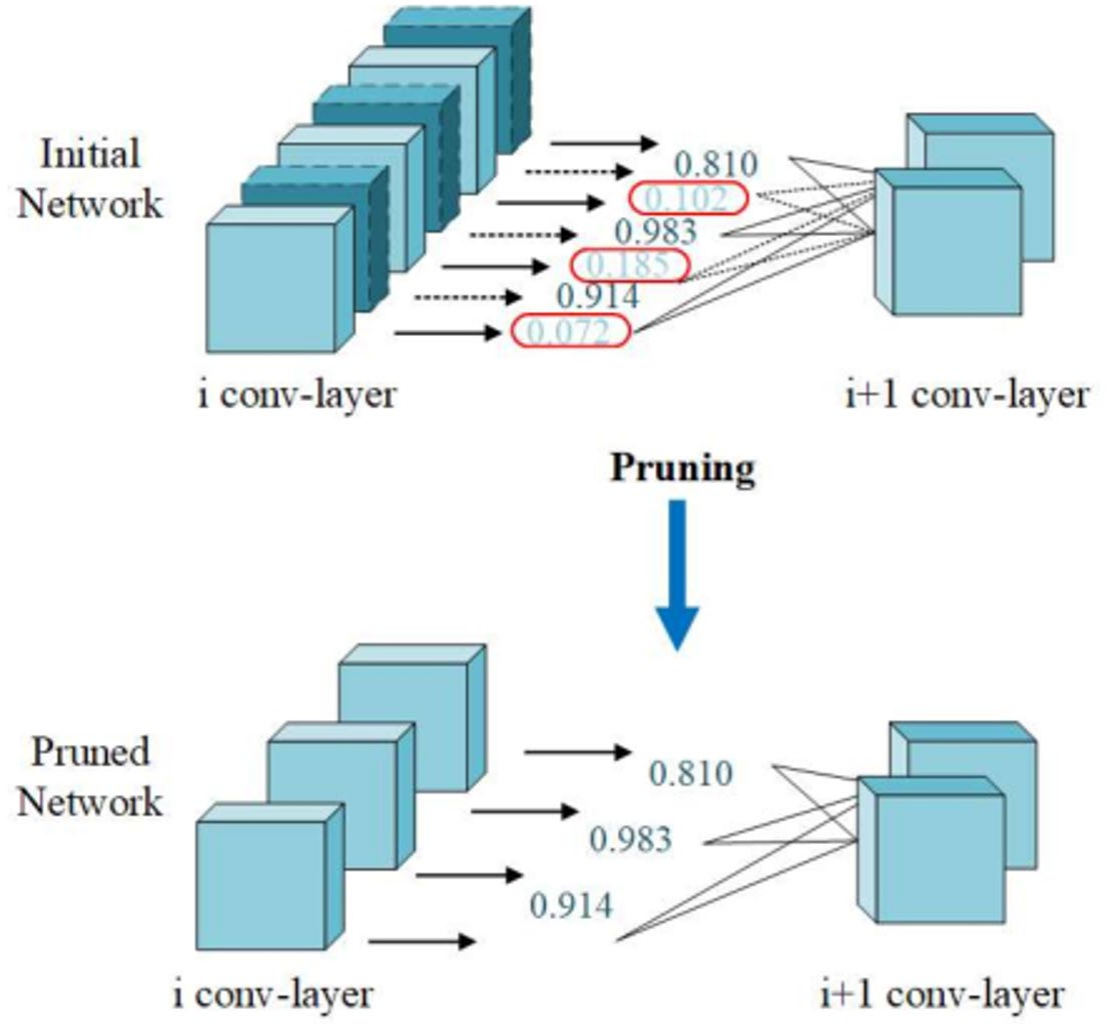
Schematic diagram of the global-local channel pruning module.

To address these issues, this study develops a global-local channel pruning module, which helps reduce the computational load by selectively pruning certain channels, or features, from the network. The calculation diagram of the global-local channel pruning module can be seen in Fig. 2. The idea behind global-local channel pruning is to balance between preserving the most informative features and maintaining the important spatial details that are crucial for accurate fracture detection. In local channel pruning, selecting channels is suitable for pruning to retain more information. This section models the problem of fine crack segmentation in high-resolution scenes as follows:

1 $$\text{argmin} _{{\beta ,W}} \frac{1}{{2N}}\left\| {Y - \sum\limits_{{i = 1}}^{c} {\beta _{i} } X_{i} W_{i}^{{ \top }} } \right\|_{F}^{2} ,\left\| \beta \right\|_{0} \le c^{\prime }$$

2 $$\text{argmin} _{{\beta ,W}} \frac{1}{{2N}}\left\| {Y - \sum\limits_{{i = 1}}^{c} {\beta _{i} } X_{i} W_{i}^{{ \top }} } \right\|_{F}^{2} + \lambda \left\| \beta \right\|_{1} ,\left\| \beta \right\|_{0} \le c^{\prime } ,\forall i\left\| {W_{i} } \right\|_{F} = 1$$

where *N* denotes the number of samples, *n* denote the Number of output channels.

Further combining the above formulas, we can simplify them to get:

3 $$\hat{\beta }^{{{\text{LASSO }}}} (\lambda ) = \text{argmin} _{\beta } \frac{1}{{2N}}\left\| {Y - \sum\limits_{{i = 1}}^{c} {\beta _{i} } Z_{i} } \right\|_{F}^{2} + \lambda \left\| \beta \right\|_{1} ,\left\| \beta \right\|_{0} \le c^{\prime }$$

The above-mentioned equations collectively model the global-local channel pruning process. The framework minimizes the reconstruction error while ensuring sparsity and maintaining key structural information. The inclusion of constraints, such as sparsity and weight normalization, ensures that the network focuses on the most relevant channels for fine crack segmentation. This optimization is particularly valuable in high-resolution scenes, where computational efficiency and accuracy are crucial, especially for real-time UAV-based crack inspection for civil infrastructures.

### The improved ResCBAM attention mechanism

**Fig. 3:**
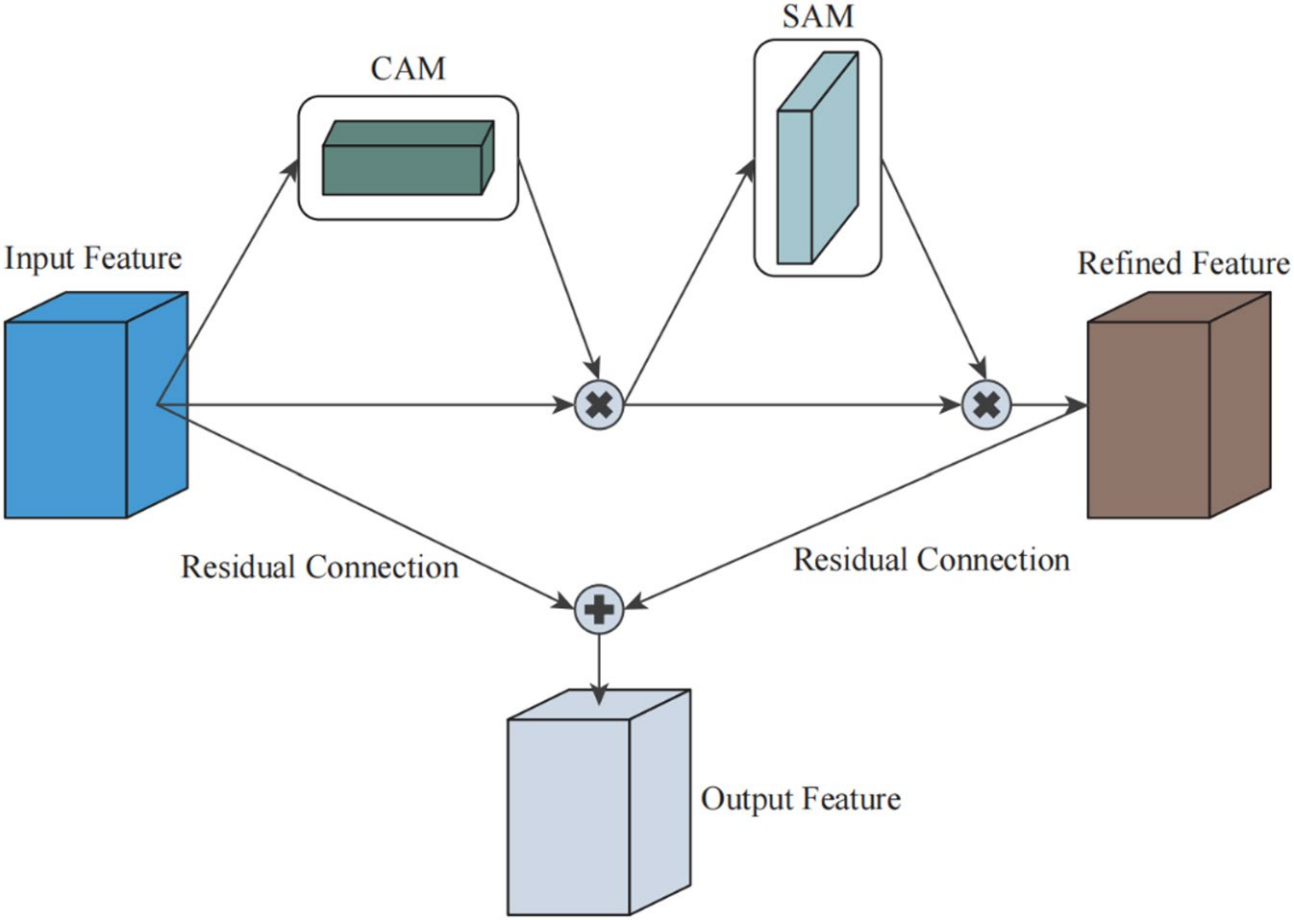
The diagram of the ResCBAM network.

The ResCBAM attention mechanism is a hybrid attention framework that integrates both residual learning and the Convolutional Block Attention Module (CBAM). Figure 3 shows the visual diagram of the ResCBAM module. The ResCBAM attention mechanism is highly feasible for detecting micro-cracks and mitigating the effects of complex noise interference in crack detection tasks. By jointly emphasizing fine-grained cues and suppressing irrelevant background signals, ResCBAM is well suited to the challenges of real-world inspection. Given adequate data quality and computational resources, it can substantially improve crack detection performance in infrastructure monitoring, where noise is prevalent and many cracks are subtle.

The formulas for the channel attention mechanism and spatial attention mechanism are as follows:

4 $$M_{{\text{c}}} (F) = \sigma \left( {W_{1} \left( {W_{0} \left( {F_{{{\text{avg }}}} } \right)} \right) + W_{1} \left( {W_{0} \left( {F_{{\max }} } \right)} \right)} \right)$$

5 $$M_{{\text{s}}} (F) = \sigma \left( {f^{{7 \times 7}} \left( {\left[ {F_{{{\text{avg }}}} ;F_{{\max }} } \right]} \right)} \right)$$

where $F_{{{\text{avg }}}}$and $F_{{\max }}$ denotes the Feature map F undergoes global average pooling and global maximum pooling; $W_{0}$ and $W_{1}$ denotes the weight matrix of the fully connected layer; $M_{{\text{c}}} (F)$ and $M_{{\text{s}}} (F)$ represents channel attention map and spatial attention map, respectively.

The main calculation process of the ResCBAM module is as follows:

#### Step 1

Apply max pooling and average pooling to the input feature map, concatenate the results, and pass them through a convolutional layer followed by a sigmoid activation to generate spatial attention weights that highlight key regions.

#### Step 2

Multiply the spatial attention weights with the original feature map to enhance informative spatial areas and suppress irrelevant or noisy regions.

#### Step 3

Apply global max pooling and average pooling across spatial dimensions, feed the resulting vectors into shared fully connected layers with sigmoid activation to produce channel-wise attention weights, and multiply them with the feature map to emphasize important channels.

### Online feature distillation and multi-feature fusion mechanism

The construction of an online feature distillation and multi-feature fusion mechanism for real-time crack detection under complex noise conditions is crucial for enhancing the robustness and accuracy of automated crack recognition systems. In real-world environments, the presence of various types of noise, such as image artifacts, varying lighting conditions, and background clutter, often degrades the performance of conventional crack detection models. Traditional methods may struggle to distinguish relevant features from irrelevant or misleading information introduced by such noise^36^.

**Fig. 4:**
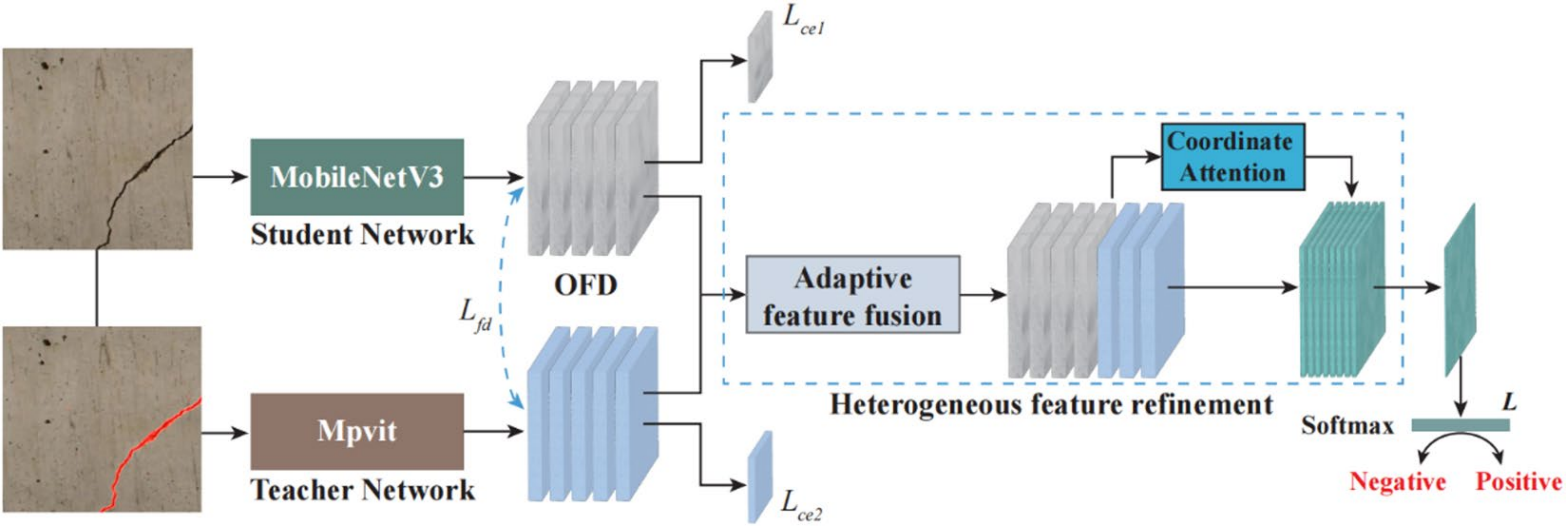
Schematic diagram of knowledge distillation and multi-feature fusion construction.

To address these limitations, an online feature distillation mechanism is proposed to select the most informative features related to cracks during the training process, allowing the model to adapt in real-time to the specific characteristics of noisy data. Figure 4 demonstrates the schematic diagram of knowledge distillation and multi-feature fusion construction. It can be inferred that the developed dynamic adjustment improves the model’s ability to focus on critical patterns associated with cracks while filtering out noise that may lead to false positives or missed detections.

Supposing that the crack pixel-wise segmentation dataset can be expressed using $X = \left\{ {x_{i} } \right\}_{{i = 1}}^{N}$. The crack image annotations can be denoted using $Y = \left\{ {y_{i} } \right\}_{{i = 1}}^{N}$. The predicted probability of the student network and teacher network$P_{c}^{m} \left( {x_{i} } \right)$ and $P_{t}^{m} \left( {x_{i} } \right)$ can be expressed using the following formulas:

6 $$P_{c}^{m} \left( {x_{i} } \right) = \frac{{\exp \left( {z_{1}^{m} } \right)}}{{\sum\limits_{{m = 1}}^{M} {\exp } \left( {z_{1}^{m} } \right)}}$$

7 $$P_{t}^{m} \left( {x_{i} } \right) = \frac{{\exp \left( {z_{2}^{m} } \right)}}{{\sum\limits_{{m = 1}}^{M} {\exp } \left( {z_{2}^{m} } \right)}}$$

where $z_{1}^{m}$denotes the output of the softmax layer in the student network, $z_{2}^{m}$ denotes the output of the softmax layer in the teacher network.

Then, in this study, the Cross-Entropy loss was used as the supervision loss, which can be denoted as follows:

8 $$L_{{ce1}} = - \sum\limits_{{i = 1}}^{N} {\sum\limits_{{m = 1}}^{M} I } \left( {y_{i} ,m} \right)\log \left( {p_{c}^{m} \left( {x_{i} } \right)} \right)$$

9 $$L_{{ce2}} = - \sum\limits_{{i = 1}}^{N} {\sum\limits_{{m = 1}}^{M} I } \left( {y_{i} ,m} \right)\log \left( {p_{t}^{m} \left( {x_{i} } \right)} \right)$$

where $L_{{ce1}}$and $L_{{ce2}}$represents the supervision loss of the teacher and the student network.

10 $$L_{{fd}} = \left\| {F_{s} - F_{t} } \right\|_{2}^{2}$$

where $F_{s}$ and $F_{t}$ represent the corresponding feature layers of the student network and the teacher network respectively.

**Fig. 5:**
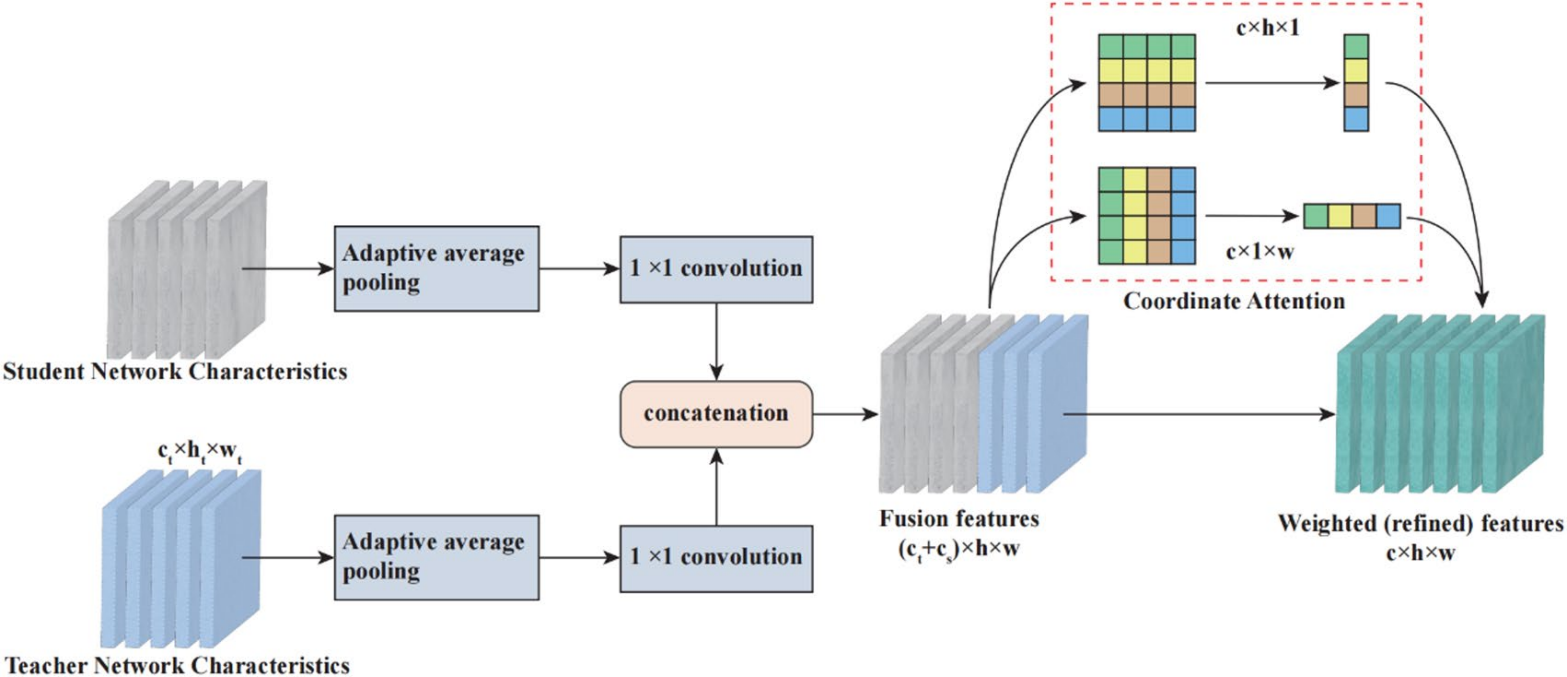
Multi-scale heterogeneous feature fusion module.

Figure 5 demonstrates the schematic diagram of the multi-scale heterogeneous feature fusion module. In summary, the student network can be guided by the implicit “dark knowledge” of the teacher network, thereby narrowing the semantic distance between heterogeneous features. Therefore, the network after distillation is used to obtain sufficient local feature information of cracks, which is convenient for constructing a fine crack segmentation model under complex noise.

## Case study

### Engineering description

**Fig. 6:**
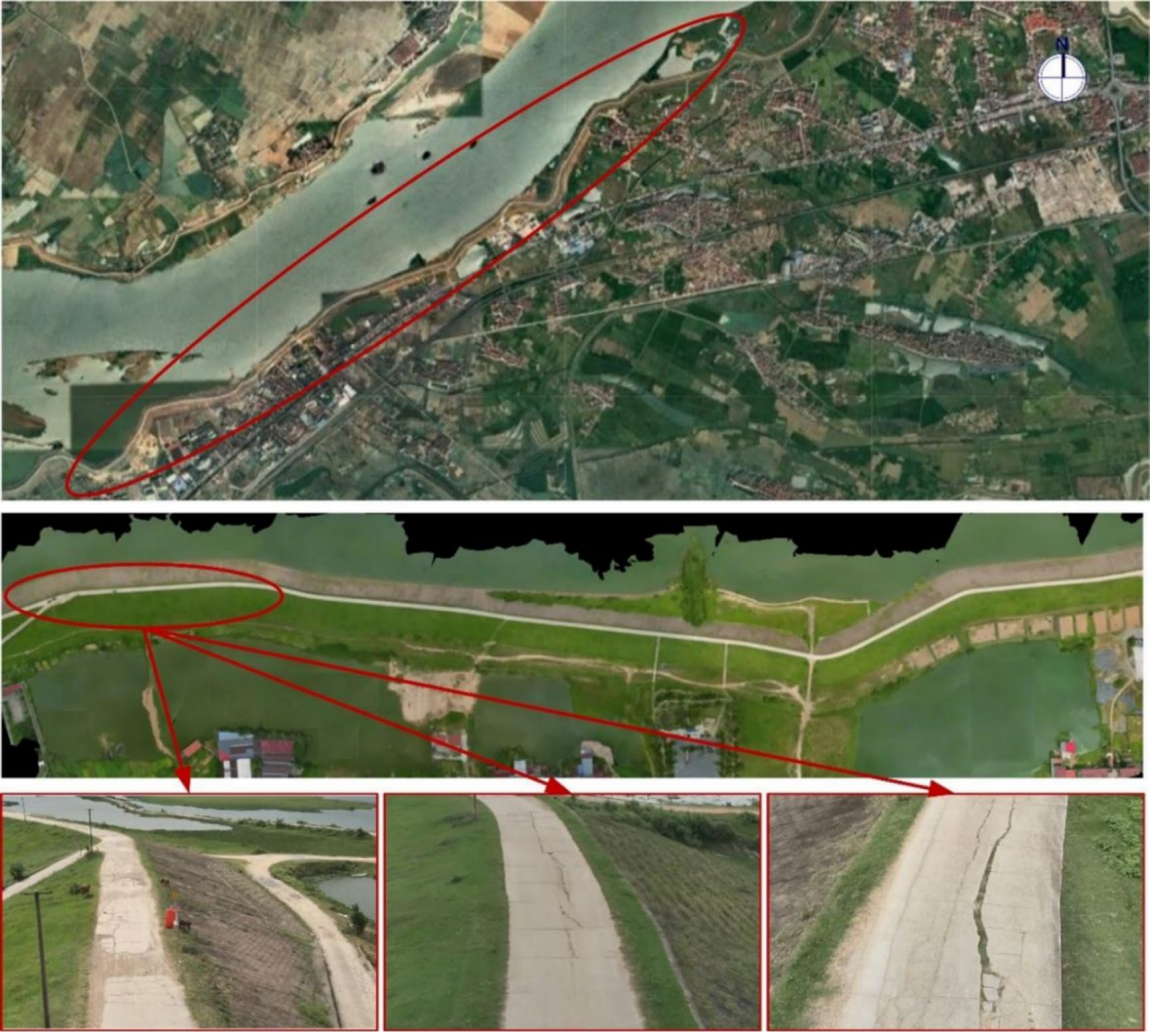
Major infrastructure in service.

The case study in this research is a long-service levee section, as illustrated in Fig. 6. The levee extends for approximately 1200 m along the riverbank, serving as a critical flood defense structure in the region. With an average height of 8 m and a crest width of 6 m, the levee is designed to resist high water levels and prolonged hydraulic loading. However, during field inspections, multiple surface cracks and localized concrete spalling were observed along the water-facing slope, raising concerns regarding the long-term stability and durability of the levee. Such defects may weaken the structural capacity of the levee to withstand extreme flood events, underscoring the need for timely assessment and effective maintenance strategies to ensure reliable flood protection.

**Fig. 7:**
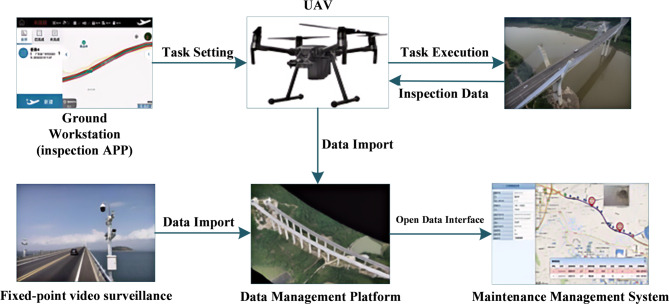
UAV systems for infrastructure inspection.

To comprehensively assess the service status of the project, a UAV-based inspection system, specifically the DJI Matrice 600 Pro, was deployed. The composition diagram of the UAV detection system is shown in Fig. 7. It can be inferred that at the core of the system is a UAV equipped with advanced payloads, including a high-resolution camera and a gimbal for stabilized imaging. The UAV receives navigational inputs via satellite-based positioning systems, ensuring precise control and accurate localization during operations. Table 1 provides a detailed overview of the technical specifications for the UAV-based inspection system. The UAV has a maximum takeoff weight of 15.5 kg, with a service ceiling that varies depending on the type of propellers used. For standard propellers, the maximum service ceiling is 2500 m above sea level, while high-altitude propellers enable a ceiling of 4500 m. The system offers a maximum flight time of approximately 32 min and a maximum hovering time of 30 min under optimal conditions. Its maximum horizontal speed is 18 m/s in the absence of wind, and it can ascend at a rate of up to 5 m/s.

**Fig. 8:**
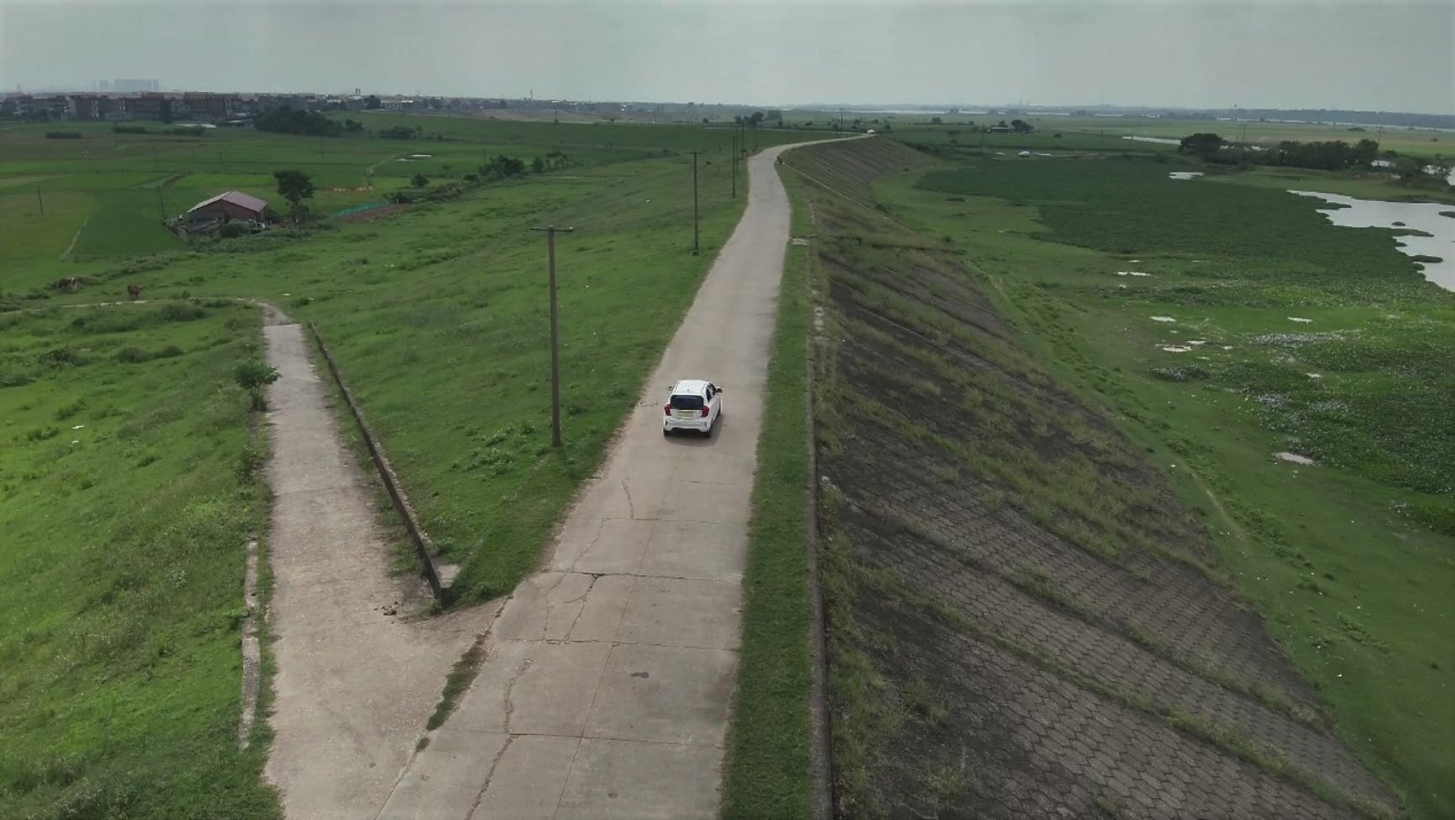
Actual UAV engineering inspection scene.

The working photos of the UAV-based inspection system applied to actual infrastructure inspection are shown in Fig. 8. It can be inferred that the UAV-based inspection system ensures real-time data acquisition, transmission, and processing to enhance the efficiency and accuracy of infrastructure monitoring. This integrated system ensures comprehensive and efficient inspection of infrastructure, reducing human risk and enabling predictive maintenance. The use of UAVs significantly improves the safety, accuracy, and cost-effectiveness of infrastructure monitoring and management.

**Table 1:** Parameter of UAV inspection systems.

Devices	Main indicator	Parameter value
UAV system	Max takeoff weight	15.5 kg
	Max service ceiling above sea level	2500 m (standard propellers); 4500 m (high-altitude propellers)
	Max flight time	Approx. 32 min
	Max hovering time	Approx. 30 min
	Max horizontal speed	18 m/s (no wind)
	Max ascend speed	5 m/s
Camera	Resolution	16 MP (4608 × 3456 pixels)
	Sensor size	17.3 × 13.0 mm
	Pixel size	3.75 μm
	Lens focal length	15 mm
	ISO range	100–25,600

### The construction of the structural damage dataset

**Fig. 9:**
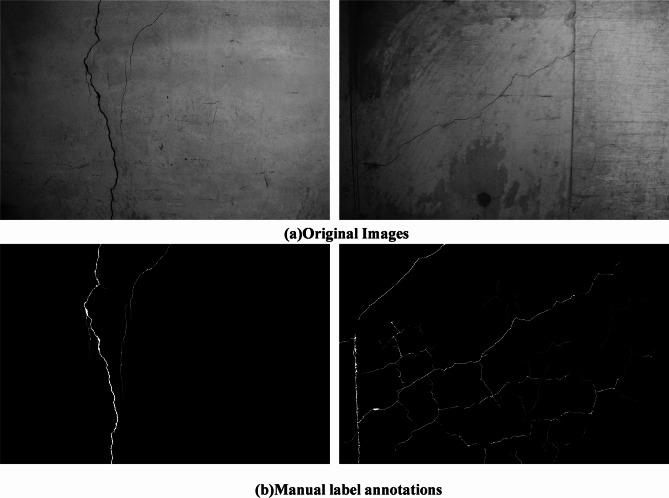
Damage images collected by UAVs and their pixel-level annotation results.

Figure 9 demonstrates the high-resolution cracks in the structure captured by the UAV inspection system. As can be seen from the figure, the concrete cracks collected by the UAV-based inspection system show typically small, narrow, and long characteristics. These cracks are generally linear or slightly curved, with widths often ranging from a few millimeters to less than a centimeter and lengths extending over several meters. The detailed imagery captured by the UAV inspection system highlights surface irregularities and fine-scale features of the cracks, making it possible to identify early-stage damage that may not be visible to the naked eye. This high-resolution data provides valuable insights into the severity and progression of structural defects, enabling precise localization and classification of cracks for further analysis and maintenance planning.

**Fig. 10:**
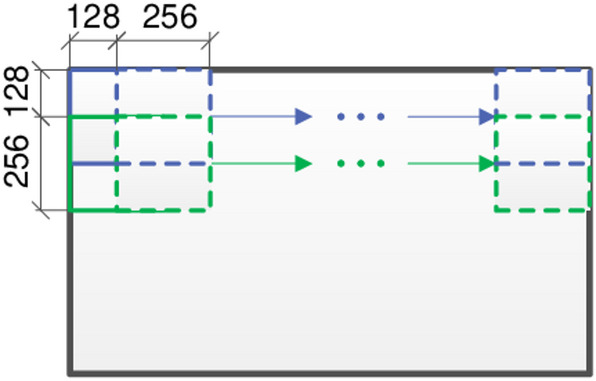
Schematic diagram of constructing damage data set using the sliding window method.

To further analyze and utilize the captured images, the sliding window method was applied to divide the high-resolution images into smaller patches. The intuitive schematic diagram of the sliding window method is shown in Fig. 10. This approach allows for the systematic segmentation of the images into manageable blocks, which were subsequently used to construct a comprehensive concrete crack dataset. The dataset serves as a valuable resource for training and testing machine learning algorithms aimed at automated crack detection and classification, enhancing the efficiency and accuracy of structural health monitoring systems.

## Experimental result and discussion

This experiment was conducted on a Windows 11 operating system. The hardware configuration includes an Intel Core i9-13900 K processor, 64 GB of memory, an NVIDIA GeForce RTX 4060 GPU, and 2 TB SSD storage. The software environment consists of Python 3.10, CUDA 12.2, and cuDNN 8.9. The deep learning framework employed in this study is PyTorch, with Visual Studio Code used as the primary platform for coding and implementation.

The training process is designed with 100 iterations and a batch size of 8. A cosine annealing learning rate schedule, a commonly used method for optimizing learning rate adjustment in deep learning models, is adopted in this study. Specifically, the initial learning rate of the model is set to 0.01. Throughout the remaining iterations, the learning rate is adjusted dynamically following a cosine function curve. This approach ensures a smooth decay of the learning rate, enhancing model convergence and reducing the risk of overfitting.

### DL-based model training process.

**Fig. 11:**
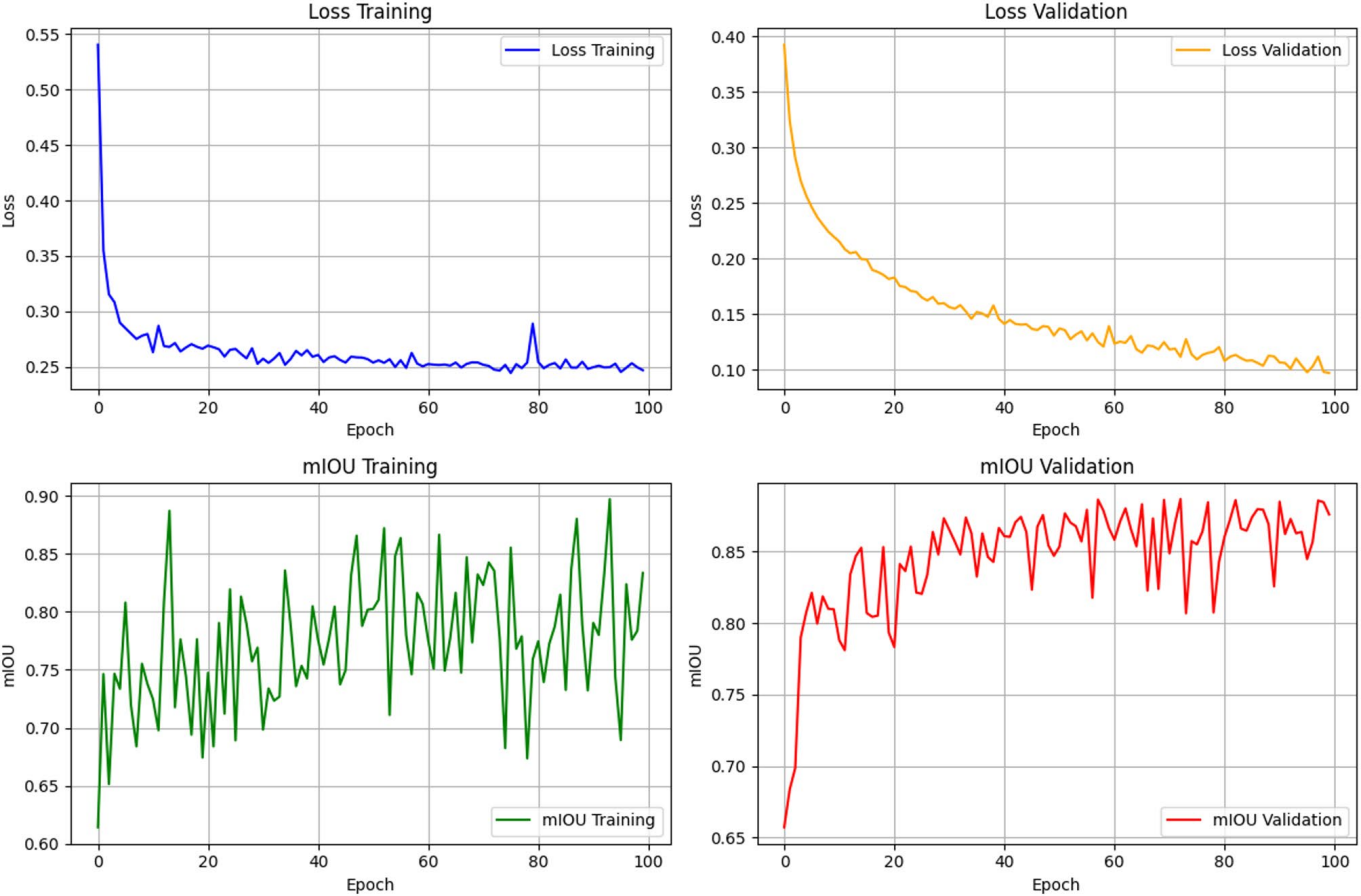
Changes in loss values ​​and evaluation indicators for training and validation sets.

Figure 11 demonstrates the training and validation performance of the proposed deep learning model over 100 epochs, focusing on loss and mIoU metrics. Both training and validation losses decrease consistently, with validation loss showing smooth convergence, indicating effective learning and generalization. The mIoU improves steadily, with training mIoU fluctuating but stabilizing between 0.80 and 0.90, while validation mIoU reaches a stable plateau above 0.85, reflecting strong model performance on unseen data. However, a spike in training loss around the 80th epoch suggests potential instability or overfitting, which may benefit from techniques like early stopping or adaptive learning rate schedules to enhance robustness and stability.

### Ablation experiment results

**Fig. 12:**
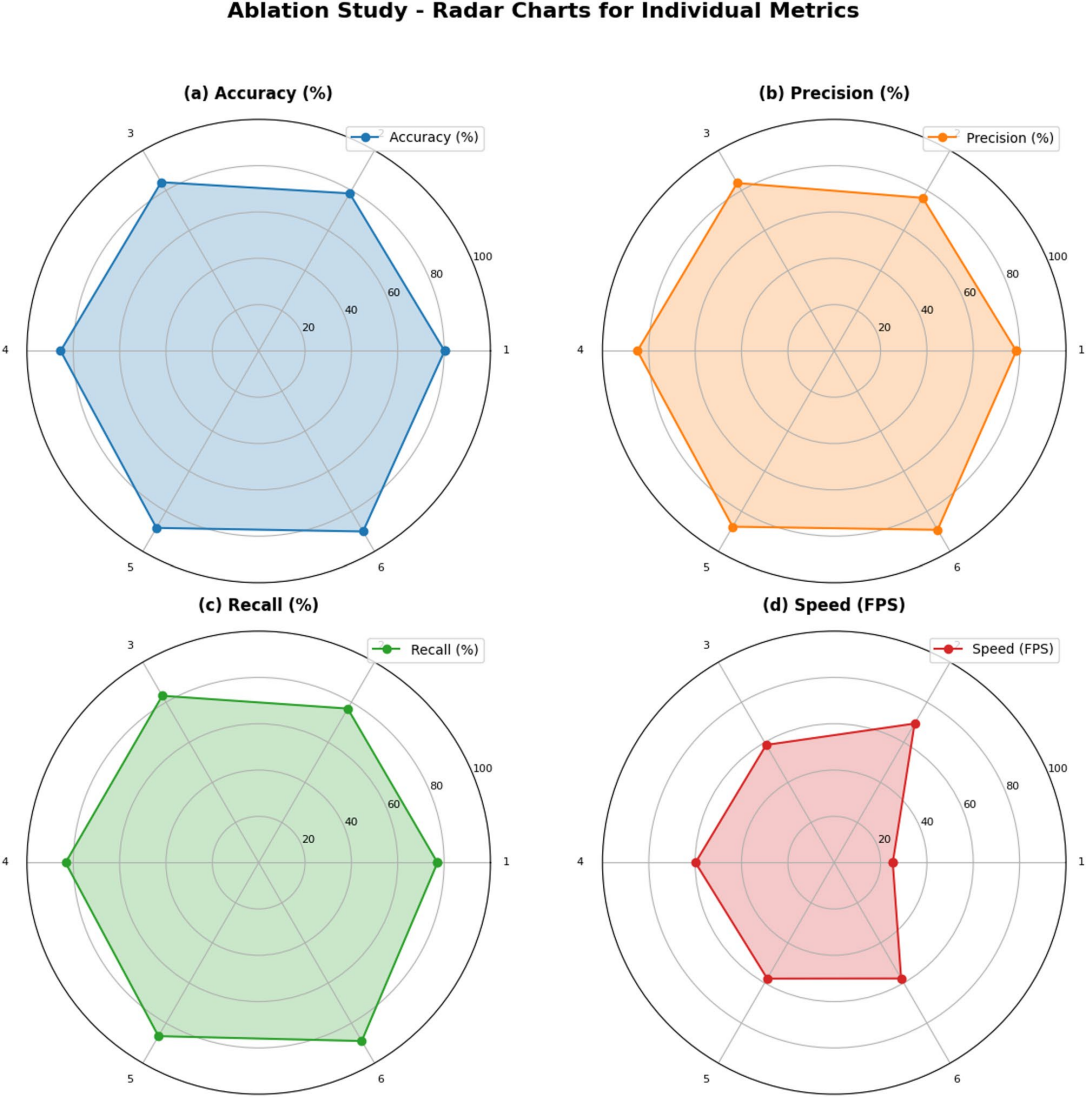
Radar chart visualization of ablation experiment results.

Table 2 demonstrates the contributions of various components in enhancing the detection performance of the proposed DL-based model. Figure 12 demonstrates the Radar chart visualization of the recognition effect of different methods in ablation experiments. It can be inferred from the figure and the table that the baseline U-Net-based model achieves moderate accuracy (80.21%), precision (78.55%), and recall (77.15%), with a relatively low inference speed of 25.25 FPS. Model pruning significantly improves computational efficiency, increasing the inference speed to 69.36 FPS(nearly about approximately 2.7 times faster) though at the expense of a slight reduction in accuracy (78.54%) and precision (76.31%). The introduction of the ResCBAM attention mechanism enhances the model’s detection capabilities in complex environments, improving accuracy, precision, and recall to 84.13%, 83.73%, and 83.22%, respectively, while maintaining reasonable efficiency (58.71 FPS). Incorporating multi-feature fusion further boosts detection performance, achieving an accuracy of 85.47% and precision of 84.94%, with minimal impact on speed (59.87 FPS). The addition of online feature distillation leads to even greater improvements, culminating in the full proposed model, which achieves the best performance across all metrics (90.05% accuracy, 89.22% precision, and 88.94% recall) while maintaining an efficient inference speed of 57.74 FPS. These experimental results indicate that the constructed DL-based model achieves an effective balance between structural damage detection accuracy and computational efficiency. The integration of model pruning, ResCBAM attention mechanism, and multi-feature fusion ensures robust performance even in noisy environments while maintaining the inference speed necessary for real-time applications. This highlights the model’s practical applicability in UAV-based structural damage inspections.

**Table 2:** Ablation experimental results of the proposed DL-based method.

Experiment	Models	Accuracy (%)	Precision (%)	Recall (%)	Speed (FPS)
1	Baseline Model	80.21	78.55	77.15	25.25
2	Baseline + Model Pruning	78.54	76.31	76.84	69.36
3	Baseline + Model Pruning + ResCBAM Attention	84.13	83.73	83.22	58.71
4	Baseline + Model Pruning + Multi-Feature fusion	85.47	84.94	83.32	59.87
5	Baseline + Model Pruning + Online feature distillation	88.25	87.65	86.51	57.81
6	Full model(Proposed)	90.05	89.22	88.94	57.74

Comparative evaluation experiment.

To further verify and evaluate the effectiveness of the DL-based model constructed in this paper in damage identification of civil infrastructure structures, state-of-the-art semantic segmentation models, including Attention U-Net^37^, RefineNet^38^, High-resolution network(HRNet)^39^, DeepLab^40^, and SegNet^41^, are introduced. Each of these models brings unique features, including attention mechanisms, multi-scale context, boundary refinement, or high-resolution representation, that can be particularly beneficial for detecting cracks in real-world infrastructure images with varying complexities and noise.

1. Attention U-Net augments the classic U-Net with attention gates that highlight salient regions (e.g., cracks) and suppress background clutter. By filtering encoder features before skip connections, it improves discrimination between damage and non-damage, boosting segmentation accuracy in noisy, real-world scenes.
2. RefineNet is a multi-path refinement architecture that progressively polishes multi-level feature maps via residual refinement modules. Its precise boundary handling makes it well-suited to fine-grained tasks such as small-crack and subtle-defect delineation.
3. HRNet maintains high-resolution representations throughout the network instead of heavily downsampling and then upsampling. This preserves fine detail while capturing global context, yielding strong performance on small cracks and localized structural damage.
4. DeepLabv3 leverages atrous (dilated) convolutions and multi-scale context aggregation to segment objects across varied scales without sacrificing spatial resolution. It is robust in cluttered environments and effective for crack patterns with wide size variability.
5. SegNet employs an encoder–decoder design that reuses max-pooling indices for efficient upsampling, lowering computation while retaining spatial detail. Its efficiency makes it attractive for real-time UAV-based crack and defect segmentation.

Table 3 demonstrates the damage identification performance comparison of different algorithms. The superior performance of the proposed method can be attributed to its innovative design and optimization strategies tailored for UAV-based real-time structural damage detection. Unlike traditional approaches, the proposed method incorporates a lightweight U-Net-based semantic segmentation network optimized through model pruning. This significantly enhances inference speed, achieving an FPS of 57.74, far surpassing other methods like HRNet (22.34) and Attention U-Net (20.23). Furthermore, the integration of an improved residual convolutional block attention module (ResCBAM) effectively addresses challenges in complex and noisy environments, such as stains and obstacles, leading to improved recall (88.94%) and IoU (88.67%) compared to HRNet and Attention U-Net. Finally, the use of online feature distillation and a multi-feature fusion mechanism ensures the effective fusion of features at different scales, enhancing the model’s detection accuracy (90.05%) and precision (89.22%) in identifying subtle structural damage. These optimizations collectively enable the proposed method to outperform existing damage detection methods in both accuracy and real-time application scenarios.

**Fig. 13:**
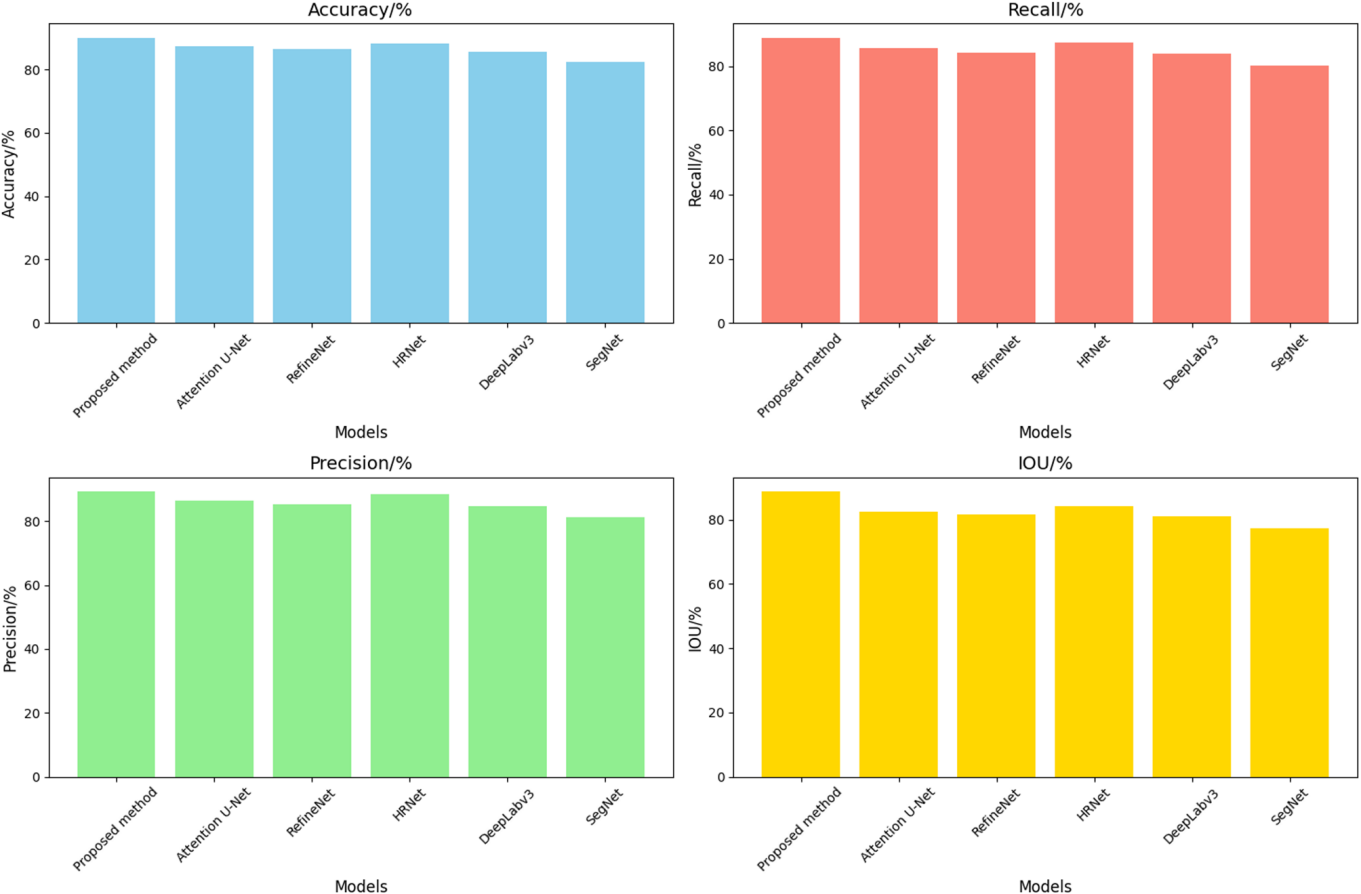
Comparison of the detection performance of the proposed and other comparison methods.

**Fig. 14:**
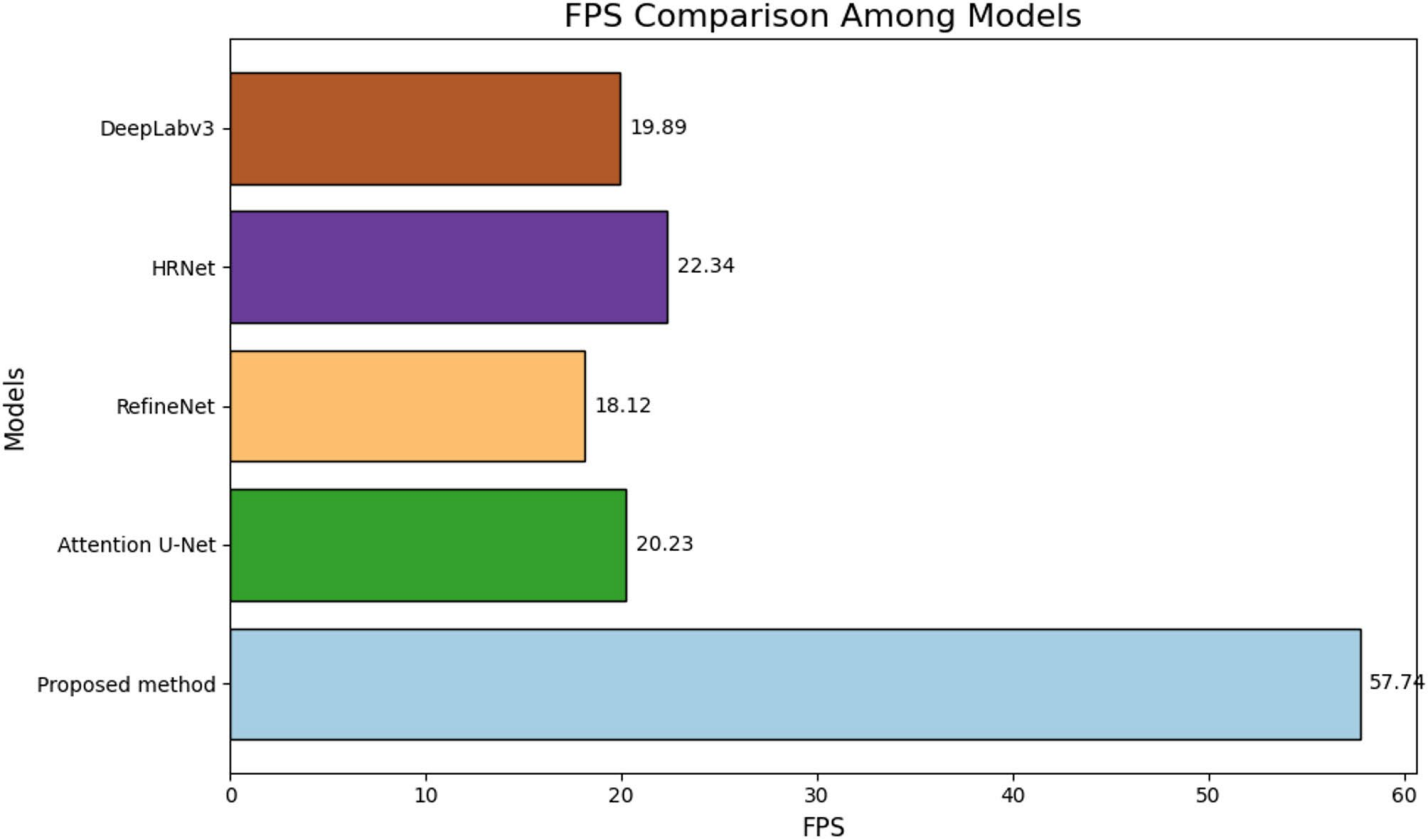
Comparison of inference efficiency between different methods.

The comparison of damage identification accuracy and inference efficiency of the proposed and other compared methods is shown in Figs. 13 and 14. The proposed method outperforms existing models across all metrics, achieving the highest accuracy (90.05%), recall (88.94%), precision (89.22%), and IoU (88.67%), while also demonstrating exceptional computational efficiency with an FPS of 57.74. Compared to HRNet and Attention U-Net, which achieve IoU values of 84.12% and 82.34% respectively, and FPS below 23, the proposed method leverages a lightweight U-Net architecture with model pruning, an improved ResCBAM for handling noisy environments, and multi-feature fusion mechanisms to enhance detection performance. These innovations enable superior damage identification accuracy and real-time applicability, making it ideal for UAV-based inspections in complex scenarios.

**Fig. 15:**
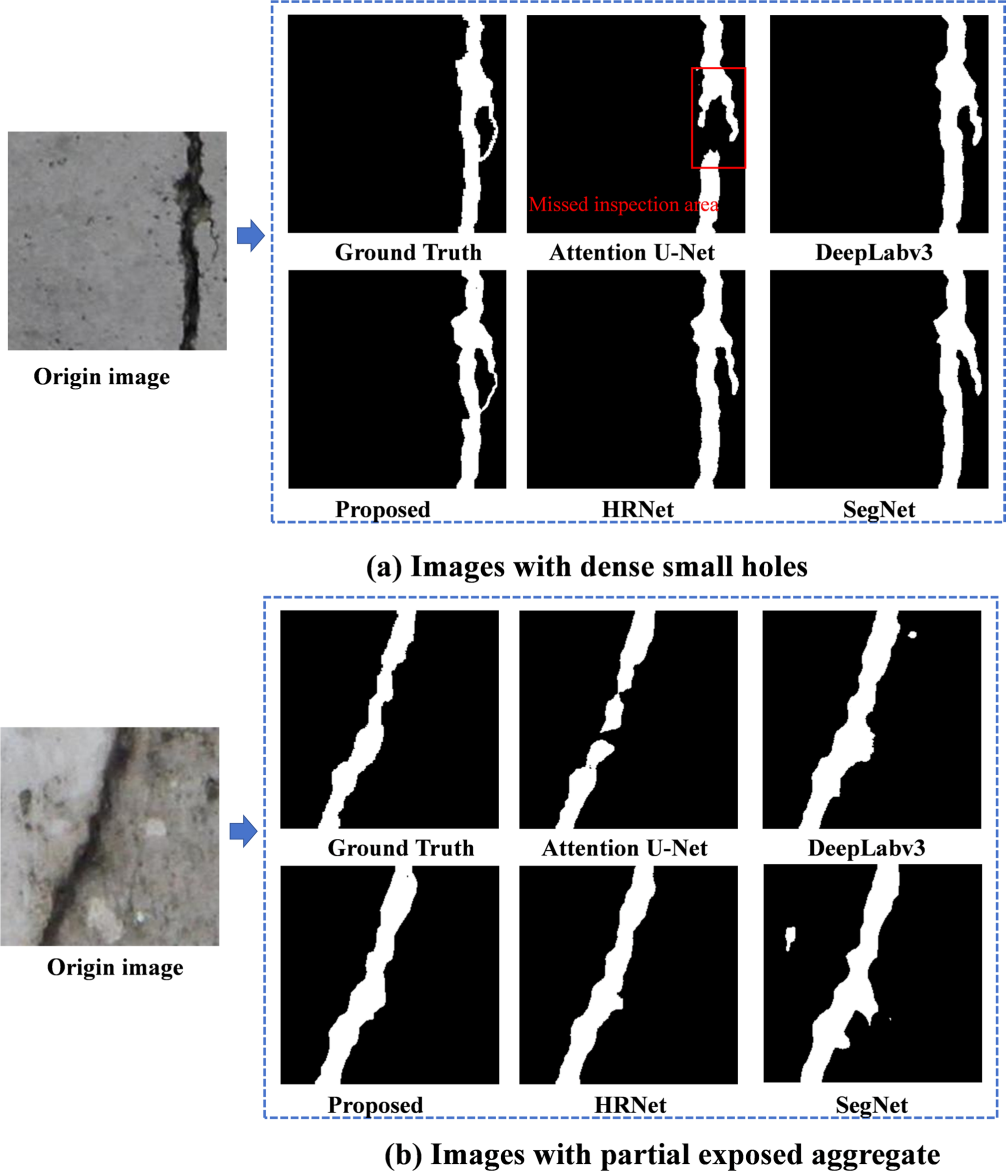
Comparison of recognition effects of different damage detection methods.

Figure 15 shows the visual comparison of damage identification results from different methods. It can be inferred that the proposed method accurately detects damaged regions even in scenarios with high noise, such as images with dense small holes. Unlike benchmark methods like attention U-Net, it minimizes false negatives, as evidenced by its ability to identify fine cracks and subtle features that are often missed. The proposed method demonstrates superior recognition performance, accurately detecting damaged regions even in high-noise scenarios. This advantage is primarily attributed to the improved ResCBAM attention mechanism, which enhances spatial and channel-wise focus on defect areas, and the online feature distillation and multi-feature fusion mechanisms, which effectively combine global contextual and fine-grained local features. These innovations enable the proposed method to minimize false negatives, precisely identify subtle cracks and defects, and maintain robustness against complex noise, outperforming all benchmark methods.

**Table 3:** Damage identification performance comparison of different algorithms.

Models	Accuracy/%	Recall/%	Precision/%	IOU/%	FPS
Proposed method	90.05	88.94	89.22	88.67	57.74
Attention U-Net	87.23	85.67	86.45	82.34	20.23
RefineNet	86.45	84.23	85.34	81.45	18.12
HRNet	88.12	87.54	88.34	84.12	22.34
DeepLabv3	85.67	83.89	84.56	80.89	19.89
SegNet	82.34	80.12	81.23	77.34	18.67

### Verification of damage recognition capability under noise interference scenarios

**Fig. 16:**
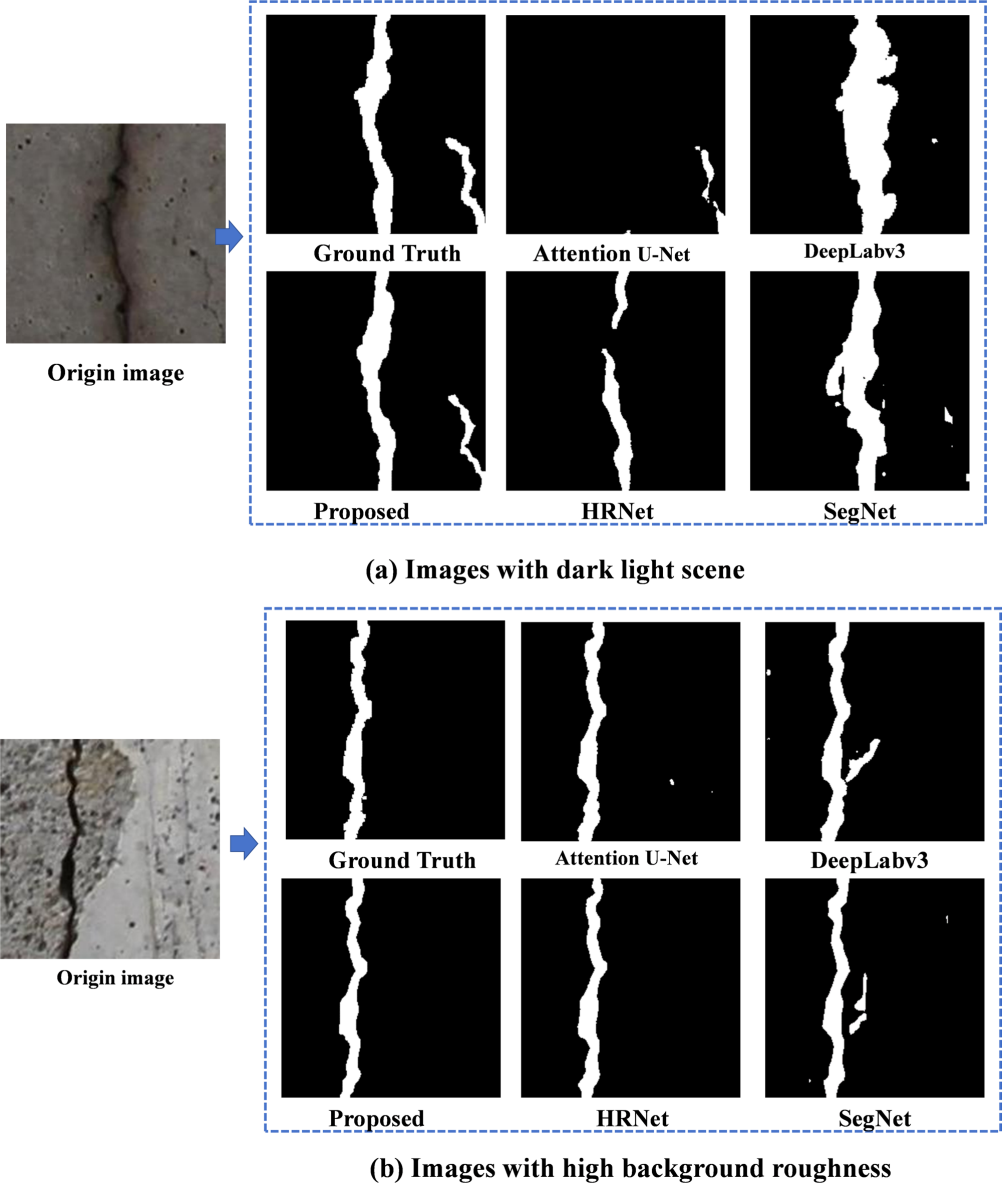
Structural damage identification results under different noises.

Figure 16 shows the comparison of structural damage identification effects under different complex noise detection conditions using the developed and benchmark methods. It can be inferred that the proposed method achieves superior recognition performance compared to the other five benchmark methods, including Attention U-Net, RefineNet, HRNet, DeepLab, and SegNet. Specifically, the proposed method demonstrates significant advantages in damage identification under challenging conditions, such as dark lighting and high background roughness. In dark light environments, where traditional methods often struggle with low contrast and reduced texture visibility, the proposed lightweight U-Net with ResCBAM and distillation method effectively extracts crack features with clear boundaries, indicating strong sensitivity to edge and texture variations. Additionally, the results highlight the model’s robustness to noise, maintaining accurate and consistent detection of cracks despite poor illumination. Under high background roughness, the proposed method exhibits superior background suppression capabilities, effectively isolating crack patterns from complex and irregular surface textures, which traditional approaches may misinterpret as noise or irrelevant features. This robustness ensures reliable damage detection across varying environmental conditions.

### Verification of defect images of different sizes

**Fig. 17:**
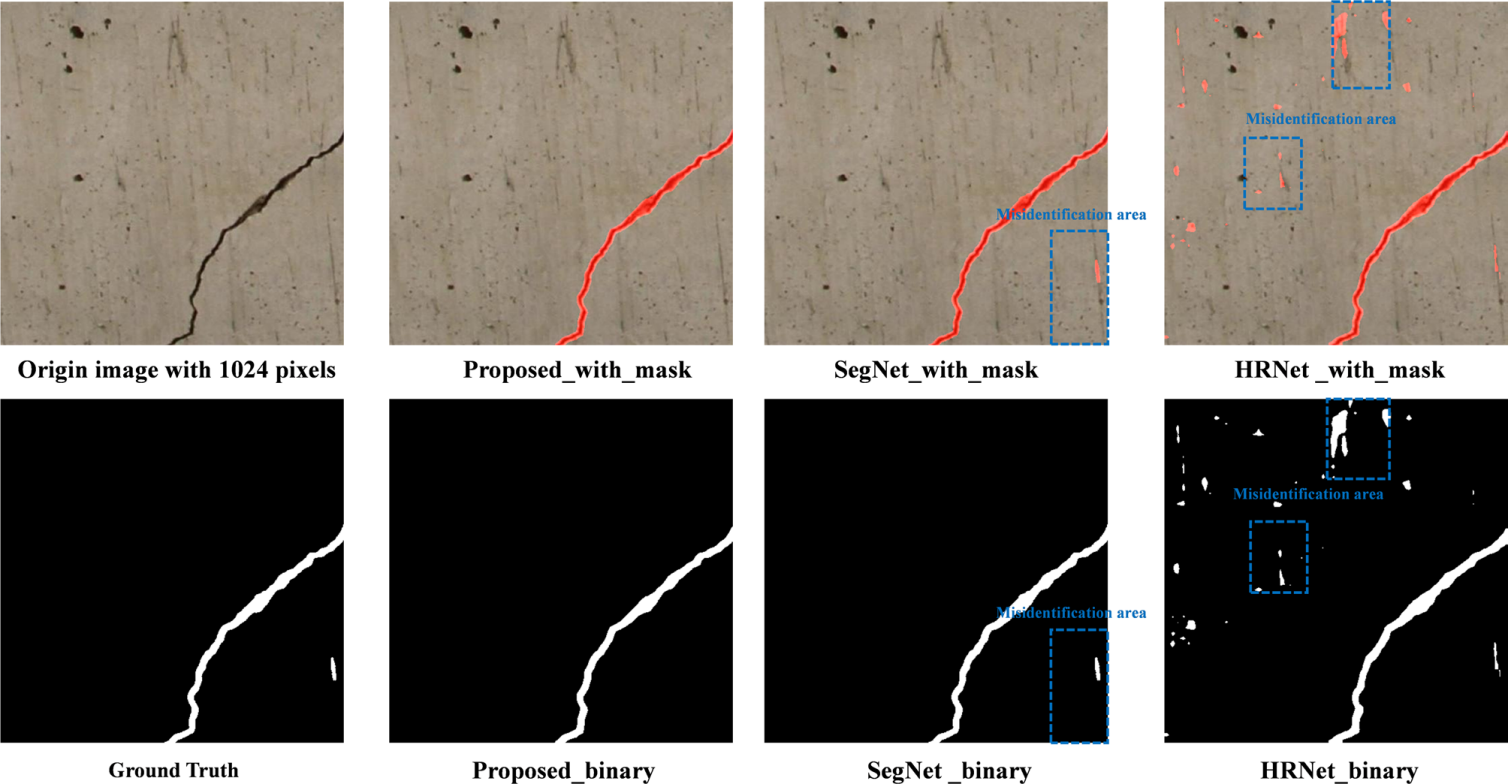
Comparison of defect recognition effects of 1024-pixel resolution UAV images.

Figure 17 demonstrates the recognition results in processing high-resolution defect images with 1024 pixels acquired by UAVs. As shown in Fig. 17, the DL-based defect recognition method proposed in this study demonstrates a remarkable ability to accurately identify and segment the geometric structure of defects. This high performance can primarily be attributed to the integration of the online knowledge distillation and multi-feature fusion mechanisms within the model architecture. The online knowledge distillation mechanism allows the proposed DL-based model to learn effectively from both high-resolution and low-resolution feature representations in real-time, enabling a more robust understanding of complex defect patterns. Simultaneously, the multi-feature fusion mechanism enhances the model’s ability to combine contextual and fine-grained features, which is crucial for detecting subtle defect details and achieving higher precision in recognition. This synergy between feature extraction and fusion not only improves the segmentation accuracy but also ensures robustness when applied to diverse defect types in high-resolution images. Furthermore, the proposed method demonstrates a strong capability to process large-scale images, effectively handling the challenges associated with high-resolution defect recognition, such as intricate defect boundaries, small-scale variations, and complex background noise. This makes the proposed method particularly suitable for UAV-based inspections, where high-resolution imagery is critical for detecting fine-grained structural defects. Overall, the results in Fig. 17 validate the efficacy and adaptability of the proposed DL-based approach in real-world applications requiring precise defect identification and segmentation.

## Conclusions and discussion

### Conclusions

Aging levee systems and escalating environmental pressures demand innovative inspection strategies to ensure long-term safety and reliability. Traditional methods are labor-intensive, costly, and limited in coverage, especially for large or hard-to-reach segments, hindering timely, comprehensive assessments. UAV-based remote sensing offers an efficient, high-resolution alternative, yet its practical value is curtailed by the lack of specialized **real-time** defect detection algorithms robust to stains, shadows, vegetation, and water reflections. To address this gap, we propose a real-time U-Net–based semantic segmentation framework tailored for UAV levee inspection. The model is optimized for high performance and deployment efficiency in noisy, complex field conditions. Ablation studies demonstrate that the ResCBAM attention module dynamically refines spatial and channel features, markedly improving detection under noise, while the multi-feature fusion mechanism integrates contextual and fine-grained cues across scales, enhancing robustness and accuracy.

These innovations collectively enable the proposed method to achieve superior performance in UAV-based levee inspections under complex environmental conditions. Comparative studies validate its advantages, attaining a detection accuracy of 90.05%, recall of 88.94%, precision of 89.22%, IoU of 88.67%, and an FPS of 57.74, substantially outperforming existing methods in both accuracy and efficiency. The high FPS rate ensures real-time applicability for large-scale levee monitoring, while the balanced precision-recall performance confirms its reliability in identifying subtle and complex defects, even in challenging scenarios involving noise, shadows, water reflections, and vegetation interference. These results underline the method’s potential for safe, precise, and efficient real-time levee health assessments, supporting proactive maintenance and flood risk reduction. Future work will focus on broadening its applicability to diverse defect categories and further enhancing computational efficiency for deployment in UAV platforms with limited onboard resources.

### Limitations and future discussion

This study primarily targets concrete defect detection for UAV-based levee inspection under complex field conditions. While UAV remote sensing enables efficient coverage of large levee surfaces, the proposed method still faces limitations in scenarios with extreme illumination, heavy occlusion (e.g., vegetation, debris), specular reflections from wet surfaces, and motion blur induced by flight dynamics. In future work, we will (i) investigate domain adaptation and synthetic-to-real transfer to improve generalization across sites, seasons, and sensors; (ii) integrate multimodal data (e.g., RGB, multispectral/thermal, LiDAR/DSM, and inertial/flight metadata) to enhance robustness against illumination and occlusion; and (iii) evaluate deployment feasibility on UAV edge devices by profiling latency, energy consumption, and thermals under real flight conditions. We also plan stress tests with controlled illumination/occlusion, uncertainty quantification to flag low-confidence predictions, and spatiotemporal modeling and multi-view/3D reconstruction to further stabilize real-time defect recognition in dynamic environments.

## Data Availability

The data that support the findings of this study are available from the corresponding author upon reasonable request.

## References

[1] Inam, H., Islam, N. U., Akram, M. U. & Ullah, F. Smart and automated infrastructure management: A deep learning approach for crack detection in Bridge images. *Sustain. Switz* **15**, (2023).

[2] Spencer, B. F., Hoskere, V. & Narazaki, Y. Advances in computer vision-based civil infrastructure inspection and monitoring. *Engineering*. **5**, 199–222 (2019).

[3] Gu, H. et al. Environmental-aware deformation prediction of water‐related concrete structures using deep learning. *Comput. -Aided Civ. Infrastruct. Eng.* **40**, 2130–2151 (2025).

[4] Liu, A., Hua, W., Xu, J., Yang, Z. & Fu, J. Concrete crack segmentation based on multi-dimensional structure information fusion-based network. *Constr. Build. Mater.* **414**, 134982 (2024).

[5] Yang, G. et al. Datasets and processing methods for boosting visual inspection of civil infrastructure: A comprehensive review and algorithm comparison for crack classification, segmentation, and detection. *Constr. Build. Mater.* **356**, 129226 (2022).

[6] Yang, Q., Shi, W., Chen, J. & Lin, W. Deep convolution neural network-based transfer learning method for civil infrastructure crack detection source task target task input input transfer. *Autom. Constr.* **116**, 103199 (2020).

[7] Bono, A., D’alfonso, L., Fedele, G., Filice, A. & Natalizio, E. Path planning and control of a UAV fleet in bridge management systems. *Remote Sens* **14**, (2022).

[8] Liu, D., Chen, J., Hu, D. & Zhang, Z. Dynamic BIM-augmented UAV safety inspection for water diversion project. *Comput. Ind.* **108**, 163–177 (2019).

[9] Tao, T. et al. Identification of ground fissure development in a semi-desert aeolian sand area induced from coal mining: utilizing UAV images and deep learning techniques. *Remote Sens* **16**, (2024).

[10] He, T., Chen, K., Jazizadeh, F. & Reichard, G. Automation in construction unmanned aerial vehicle-based as-built surveys of buildings. *Autom. Constr.* **161**, 105323 (2024).

[11] Zhao, Y., Lu, B. & Alipour, M. Automation in construction optimized structural inspection path planning for automated unmanned aerial systems. *Autom. Constr.* **168**, 105764 (2024).

[12] Chen, I. H., Ho, S. C. & Su, M. B. Computer vision application programming for settlement monitoring in a drainage tunnel. *Autom. Constr.* **110**, 103011 (2020).

[13] Fang, W., Love, P. E. D., Luo, H. & Ding, L. Computer vision for behaviour-based safety in construction: A review and future directions. *Adv. Eng. Inf.* **43**, 100980 (2020).

[14] Liu, Z. et al. Automatic intelligent recognition of pavement distresses with limited dataset using generative adversarial networks. *Autom. Constr.* **146**, 104674 (2023).

[15] Wu, J., Ye, Y. & Du, J. Automation in construction multi-objective reinforcement learning for autonomous drone navigation in urban areas with wind zones. *Autom. Constr.* **158**, 105253 (2024).

[16] Wang, R., Chen, R. Q., Guo, X. X., Liu, J. X. & Yu, H. Y. Automatic recognition system for concrete cracks with support vector machine based on crack features. *Sci. Rep* **14**, (2024). 10.1038/s41598-024-71075-1 PMC11362527 39209999

[17] Yoon, J., Lee, J., Kim, G., Ryu, S. & Park, J. Deep neural network-based structural health monitoring technique for real-time crack detection and localization using strain gauge sensors. *Sci. Rep.* **12**, (2022). 10.1038/s41598-022-24269-4 PMC9684428 36418390

[18] Li, G., Li, X., Zhou, J., Liu, D. & Ren, W. Pixel-level bridge crack detection using a deep fusion about recurrent residual convolution and context encoder network. *Meas. J. Int. Meas. Confed*. **176**, 109171 (2021).

[19] Zhang, Y. & Yuen, K. V. Crack detection using fusion features-based broad learning system and image processing. *Comput. -Aided Civ. Infrastruct. Eng.* **36**, 1568–1584 (2021).

[20] Zhang, F. et al. A new identification method for surface cracks from UAV images based on machine learning in coal mining areas. *Remote Sens.* **12**, (2020).

[21] Duan, Z., Liu, J., Ling, X., Zhang, J. & Liu, Z. ERNet: A rapid road crack detection method using Low-Altitude UAV remote sensing images. *Remote Sens.* **16**, (2024).

[22] Fan, Q. et al. A novel high-precision lightweight concrete Bridge crack image recognition method based on knowledge distillation. (2024). 10.1177/14759217241274277.

[23] Li, Y. et al. Vision-guided crack identification and size quantification framework for dam underwater concrete structures. *Struct. Health Monit.* **24**, 2125–2148 (2025).

[24] Zhou, R., Almustafa, M. K., Nehdi, M. L. & Su, H. Automated localization of dike leakage outlets using UAV-Borne thermography and YOLO-based object detectors. *ISPRS J. Photogramm. Remote Sens.* **218**, 551–573 (2024).

[25] Xu, Y., Fan, Y. & Li, H. Lightweight semantic segmentation of complex structural damage recognition for actual bridges. *Struct. Health Monit.* **22**, 3250–3269 (2023).

[26] Ye, X. et al. An advanced AI-based lightweight two-stage underwater structural damage detection model. *Adv. Eng. Inf.* **62**, 102553 (2024).

[27] Wang, W., Su, C., Han, G. & Zhang, H. A lightweight crack segmentation network based on knowledge distillation. *J. Build. Eng.* **76**, 107200 (2023).

[28] Zhang, J., Wang, Z., Chen, J., Wang, F. & Gao, L. An automated framework for abnormal target segmentation in levee scenarios using fusion of UAV-based infrared and visible imagery. *Remote Sens.* **17**, 3398MDPI (2025).

[29] Wu, X. et al. Gravity dam displacement monitoring using in situ strain and deep learning. *Comput. -Aided Civ. Infrastruct. Eng.* **40**, 348–368 (2025).

[30] Ma, Y. et al. GANFormerNet: A UAV-based concrete crack segmentation model for water-related structures using vision transformer and graph attention network. *Adv. Eng. Inf.* **68**, 103725 (2025).

[31] Zhou, L. et al. UAV vision-based crack quantification and visualization of bridges: system design and engineering application. *Struct. Health Monit.* **24**, 1083–1100 (2025).

[32] An, S. & Rui, X. A. High-precision water body extraction method based on improved lightweight U-Net. *Remote Sens.* **14**, (2022).

[33] Litjens, G. et al. A survey on deep learning in medical image analysis. *Med. Image Anal.* **42**, 60–88 (2017). 28778026 10.1016/j.media.2017.07.005

[34] Cheng, H., Zhang, M. & Shi, J. Q. A survey on deep neural network pruning: Taxonomy, comparison, analysis, and recommendations. *IEEE Trans. Pattern Anal. Mach. Intell.* **46**, 10558–10578 (2024). 39167504 10.1109/TPAMI.2024.3447085

[35] Mao, J. et al. Attention map guided transformer pruning for occluded person re-identification on edge device. *IEEE Trans. Multimed*. **25**, 1592–1599 (2023).

[36] Zheng, J., Chen, L., Wang, J., Chen, Q. & Huang, X. Automation in construction knowledge distillation with T-Seg guiding for lightweight automated crack segmentation. *Autom. Constr.* **166**, 105585 (2024).

[37] Oktay, O. et al. Attention u-net: Learning where to look for the pancreas. *ArXiv Prepr. ArXiv180403999* (2018).

[38] Lin, G., Milan, A., Shen, C., Reid, I. & Refinenet Multi-path refinement networks for high-resolution semantic segmentation. In *Proceedings of the IEEE Conference on Computer Vision and Pattern Recognition* 1925–1934 (2017).

[39] Wang, J. et al. Deep high-resolution representation learning for visual recognition. *IEEE Trans. Pattern Anal. Mach. Intell.* **43**, 3349–3364 (2020). 10.1109/TPAMI.2020.2983686 32248092

[40] Chen, L. C. C. et al. Semantic image segmentation with deep convolutional nets, atrous convolution, and fully connected Crfs. *IEEE Trans. Pattern Anal. Mach. Intell.* **40**, 834–848 (2017). 28463186 10.1109/TPAMI.2017.2699184

[41] Badrinarayanan, V., Kendall, A. & Cipolla, R. Segnet: A deep convolutional encoder-decoder architecture for image segmentation. *IEEE Trans. Pattern Anal. Mach. Intell.* **39**, 2481–2495 (2017). 28060704 10.1109/TPAMI.2016.2644615

